# Genome-wide identification of genic and intergenic neuronal DNA regions bound by Tau protein under physiological and stress conditions

**DOI:** 10.1093/nar/gky929

**Published:** 2018-10-13

**Authors:** Houda Benhelli-Mokrani, Zeyni Mansuroglu, Alban Chauderlier, Benoit Albaud, David Gentien, Sabrina Sommer, Claire Schirmer, Lucie Laqueuvre, Thibaut Josse, Luc Buée, Bruno Lefebvre, Marie-Christine Galas, Sylvie Souès, Eliette Bonnefoy

**Affiliations:** 1 Université Paris Descartes, Centre Interdisciplinaire Chimie Biologie-Paris, Inserm UMRS1007, Paris, France; 2 Université de Lille, Institut National de la Santé et de la Recherche Medicale (INSERM), CHU Lille, UMR-S 1172, LabEx DISTALZ, Alzheimer & Tauopathies, Lille, France; 3 Institut Curie, PSL Research University, Translational Research Departement, Genomics Platform, Paris, F-75248 France; 4 Université François Rabelais, Institut de Recherche sur la Biologie de l’Insecte, CNRS UMR 7261, Tours, France

## Abstract

Tauopathies such as Alzheimer's Disease (AD) are neurodegenerative disorders for which there is presently no cure. They are named after the abnormal oligomerization/aggregation of the neuronal microtubule-associated Tau protein. Besides its role as a microtubule-associated protein, a DNA-binding capacity and a nuclear localization for Tau protein has been described in neurons. While questioning the potential role of Tau-DNA binding in the development of tauopathies, we have carried out a large-scale analysis of the interaction of Tau protein with the neuronal genome under physiological and heat stress conditions using the ChIP-on-chip technique that combines Chromatin ImmunoPrecipitation (ChIP) with DNA microarray (chip). Our findings show that Tau protein specifically interacts with genic and intergenic DNA sequences of primary culture of neurons with a preference for DNA regions positioned beyond the ±5000 bp range from transcription start site. An AG-rich DNA motif was found recurrently present within Tau-interacting regions and 30% of Tau-interacting regions overlapped DNA sequences coding for lncRNAs. Neurological processes affected in AD were enriched among Tau-interacting regions with *in vivo* gene expression assays being indicative of a transcriptional repressor role for Tau protein, which was exacerbated in neurons displaying nuclear pathological oligomerized forms of Tau protein.

## INTRODUCTION

Tauopathies are neurodegenerative disorders associated to the presence, in neurons, of abnormal forms of the microtubule associated Tau protein (1–3). Over 20 different tauopathies are presently described (4) with Alzheimer's Disease (AD) being the most common, affecting >36 million people world-wide. Age-related cognitive dysfunction with memory loss is the prevailing symptom of tauopathies for which there is presently no cure (5).

Tau is an intrinsically disordered protein (6) that was first identified as a microtubule associated protein (MAP) of the brain with a predominant axonal distribution (2). It has been shown to play a role in the polymerization of tubulins, the dynamic assembly of microtubules as well as the transport along microtubules (7). The appearance of pathological hyper-phosphorylated and oligomerized/aggregated forms of Tau protein is accompanied by an abnormal localization of the protein in the somatodendritic compartment correlated with a high degree of synaptic dysfunction and loss (2,8). Notwithstanding its predominant role as a MAP, other functions for Tau protein have been described (9): in relation with genomic stability (10), protection from DNA and RNA damage induced by heat and oxidative stress (11–13), and dense chromatin structures maintenance (14,15).

Even though a causal relationship has been established between Tau dysfunction and neurodegeneration, the etiology of most tauopathies remains to be deciphered and thus, despite several decades of worldwide research on AD, efficient therapeutic solutions are still missing (5,16). Until recently, most of the research on Tau protein in relation to the development of neurodegenerative disorders has been focused on the role of Tau protein as a MAP. However, in front of the present lack of therapeutic strategies, there is a need to explore the non-MAP functions of Tau protein and their potential implications in the etiology of tauopathies (3,5). Previous research from our group as well as others, working in the field of tauopathies, has shown (i) a nuclear distribution for Tau protein in neuronal and non-neuronal cells (11,17,18), (ii) that Tau protein binds nucleic acids and forms protein-DNA complexes (11,19–21), (iii) that gene expression deregulation accompanies the development of tauopathies (22–26) and (iv) modifications in chromatin architecture and genome organization in the AD brain (27). In this context, we have questioned in this work the capacity of Tau protein to interact with specific DNA sequences of the neuronal genome, and eventually affect the expression of the associated genes. Using genome wide chromatin immunoprecipitation followed by microarray hybridization (ChIP-on-chip) assays, we have identified in this work a set of specific DNA genomic sequences of differentiated neurons from murine primary neuronal cultures of embryonic origin as significantly bound by Tau protein. Tau-interacting DNA regions identified in this study were positioned in genic as well as intergenic sequences. A preference of Tau protein for DNA regions overlapping with DNA sequences coding for long non-coding (lnc)RNAs as well as for DNA regions comprising an AG-rich motif was observed. Genic Tau-interacting DNA regions appeared functionally related with neurological functions and diseases. *In vivo* expression analysis of a subset of Tau-interacting genes performed in brain from wild type (WT), Tau-deficient (KOTau) and THY-Tau22, a transgenic model of AD-like tauopathy (28), mice revealed a role for Tau protein in repressing the expression of Tau-interacting genes specially in the context of stress-induced gene-expression. The transcriptional repressor role of Tau protein was abnormally exacerbated in neurons from the hippocampal CA1 region of THY-Tau22 mice displaying a nuclear accumulation of pathological oligomerized forms of Tau protein.

## MATERIALS AND METHODS

### Primary neuronal cultures and RNA collection

Wild-type and knock-out Tau (KOTau) mouse (29) primary cultures of cortical neurons were prepared from 15 to 17 day-old-embryo as described previously (30) and carried out in accordance with the approved guidelines. Briefly, embryonic cortex (from 8 to 10 embryos *per* culture) was carefully dissected and mechanically dissociated in culture medium by triturating with a polished Pasteur pipette. After 10 days *in vitro* (DIV), cells were either maintained at 37°C (control conditions) or exposed to 44°C (heat stress, HS conditions) in 5% CO_2_ incubator for 1 h as previously described (11). Cells were gently washed with PBS at 37°C and then scratched in lysis buffer from QIAGEN (Kit#74136). Total RNAs were extracted according to the manufacturer instruction and stored at −80°C. RNA concentration and purity (A260/A280) were determined using nanodrop.

### ChIP-on-chip experiments

Chromatin immunoprecipitation followed by microarray hybridization (ChIP-on-chip) experiments were performed on genomic DNA prepared from three independent cultures of neurons of murine embryonic origin cultured under either physiological or heat-stress (HS) conditions. Chromatin immunoprecipitation experiments were performed as previously described (31). For each experiment, 10 μg of sonicated genomic DNA were immunoprecipitated with either anti-Tau antibody (Tau1) directed against Tau unphosphorylated at epitope 195–202 (MAB3420 from Pierce) or, as a negative control, an antibody directed against the viral protein NSs encoded by Rift Valley Fever Virus (31) using Protein A-Agarose/Salmon Sperm DNA beads from Upstate. After elution from the beads, immunoprecipitated DNA was tagged with primer A (5′-GTTTCCCAGTCACGGTC(NNNNNNNN)-3′) that carries eight random nucleotides (N), using the DNA sequenase from USB/Affymetrix, then tagged DNA was amplified using Taq polymerase and primer B (5′-GTTTCCCAGTCACGGTC-3′) under linear conditions. Labelling of the linearly amplified DNA, hybridization on Affymetrix Mouse Promoter 1.0R arrays and array scanning were conducted at the Department of Translational Research of Institut Curie (France). Data from .cel files (GCOS 1.3 software) were imported into the Partek Genomic Suite (PGS) Software, Partek Inc., St Louis, MO, USA. Analysis was done using the tiling regulation study in PGS protocols as described in the results section. The list of Tau1 versus anti-NSs enriched regions, under physiological and HS conditions, was used to interrogate RefSeq transcripts data base to find overlapping genes and to classify regions as genic that is from 5000 base pairs (bp) upstream of Transcription Start Site (TSS) to 5000 bp downstream of STOP codon or as intergenic. The list of: (i) the genic DNA regions interacting with Tau protein under physiological conditions (Supplementary Table S1); (ii) the gene section classification of all DNA regions interacting with Tau protein under physiological conditions (Supplementary Table S2) and (iii) the list of genic DNA regions interacting with Tau protein under HS conditions (Supplementary Table S3) are available on line at http://microarrays.curie.fr/publications/UMRS1007/Benhelli-Mokrani_2018/

### Analysis of PGS data

**Figure 1.**
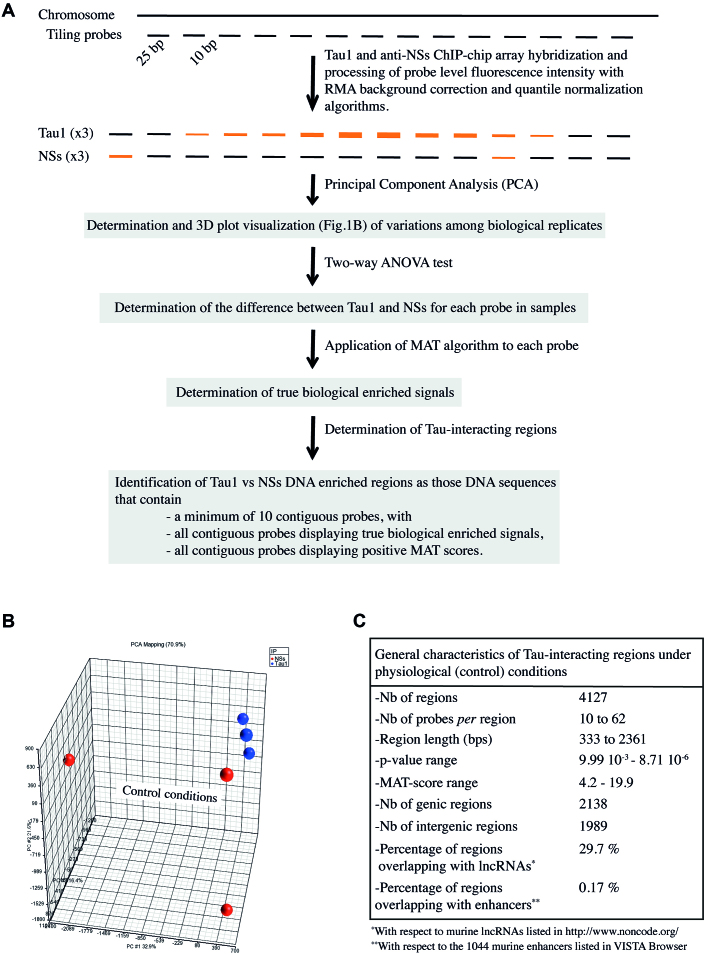
Identification of Tau-interacting regions under physiological conditions by ChIP-on-chip. (**A**) Schematic diagram describing the different steps of the analysis of results of ChIP-on-chip assays carried out after immunoprecipitation of the genome of murine neurons with Tau1 and anti-NSs antibodies. Analysis was carried out using the PGS software that uses MAT algorithm to detect significantly enriched DNA sequences. (**B**) Principal component analysis (PCA) plot that separates samples according to abundance variation showing that the Tau1 samples cluster tightly (little variation) compared to non-specific anti-NSs samples that displayed strong variation. (**C**) General characteristics of DNA regions identified as significantly enriched in Tau1 compared to anti-NSs samples, considered as Tau-interacting regions. The *P*-value of the region corresponds to the empirical *P*-value (as determined using a two-way ANOVA test) of the most significant probe MAT score included within this region and the MAT score of the region is the maximum probe MAT score for this region. The *P*-values and MAT scores of Tau-interacting regions are indicated in Supplementary Table S1. Positive MAT scores: Tau1 enriched compared to anti-NSs.

**Figure 2.**
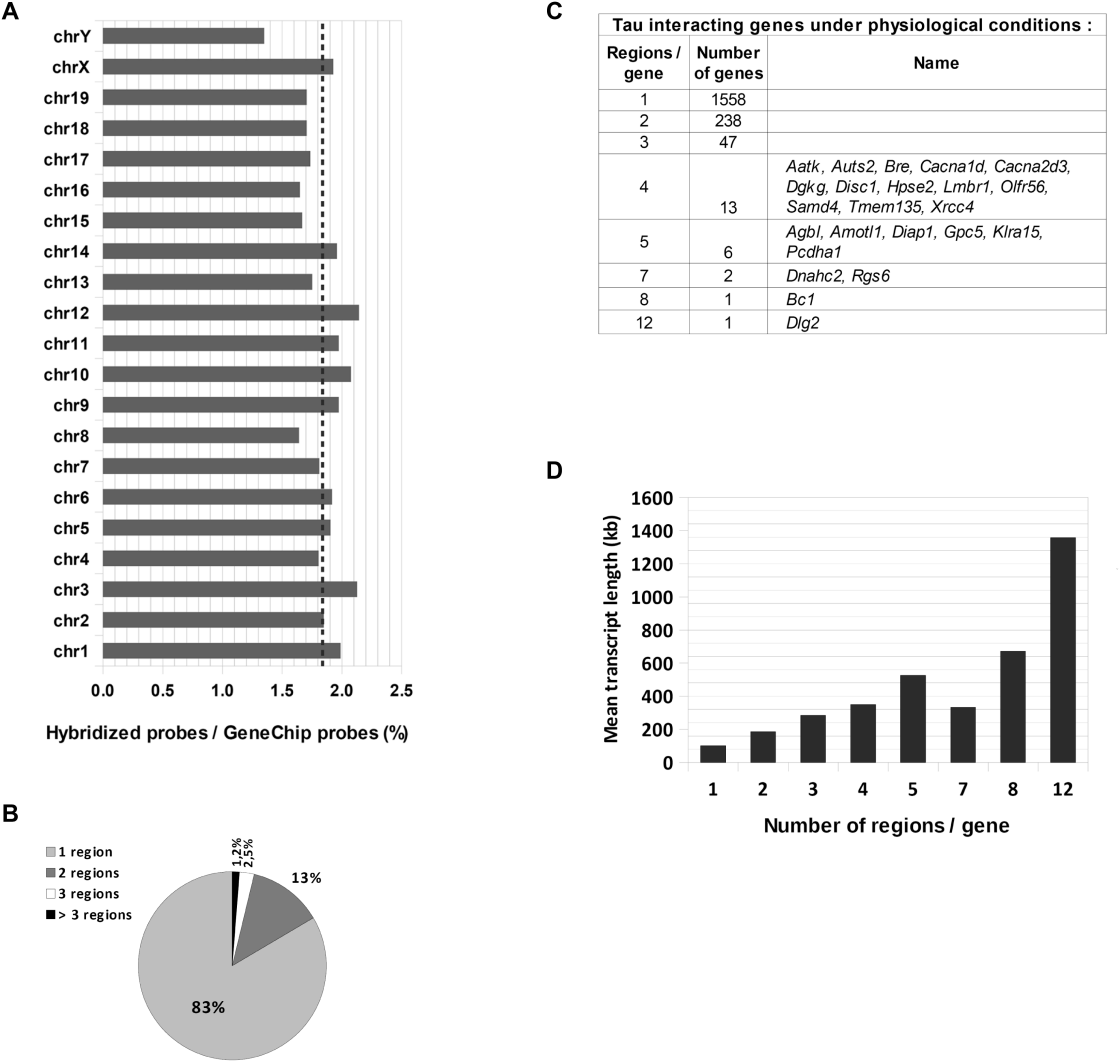
Tau protein interacts with neuronal DNA sequences under physiological conditions. (**A**) Distribution among the chromosomes of the DNA probes bound by Tau protein, expressed as a percentage of the total number of probes per chromosome present in the array; the mean value is indicated by a dashed line. (**B**) Percentage of the 1866 annotated Tau-interacting genes displaying 1, 2, 3 or >3 Tau-interacting regions. (**C**) Number of genes displaying 1 to 12 Tau-interacting regions and list of those displaying 4 and more. (**D**) Number of Tau-interacting regions per gene according to the mean length (in kb) of the corresponding transcripts.

**Figure 3.**
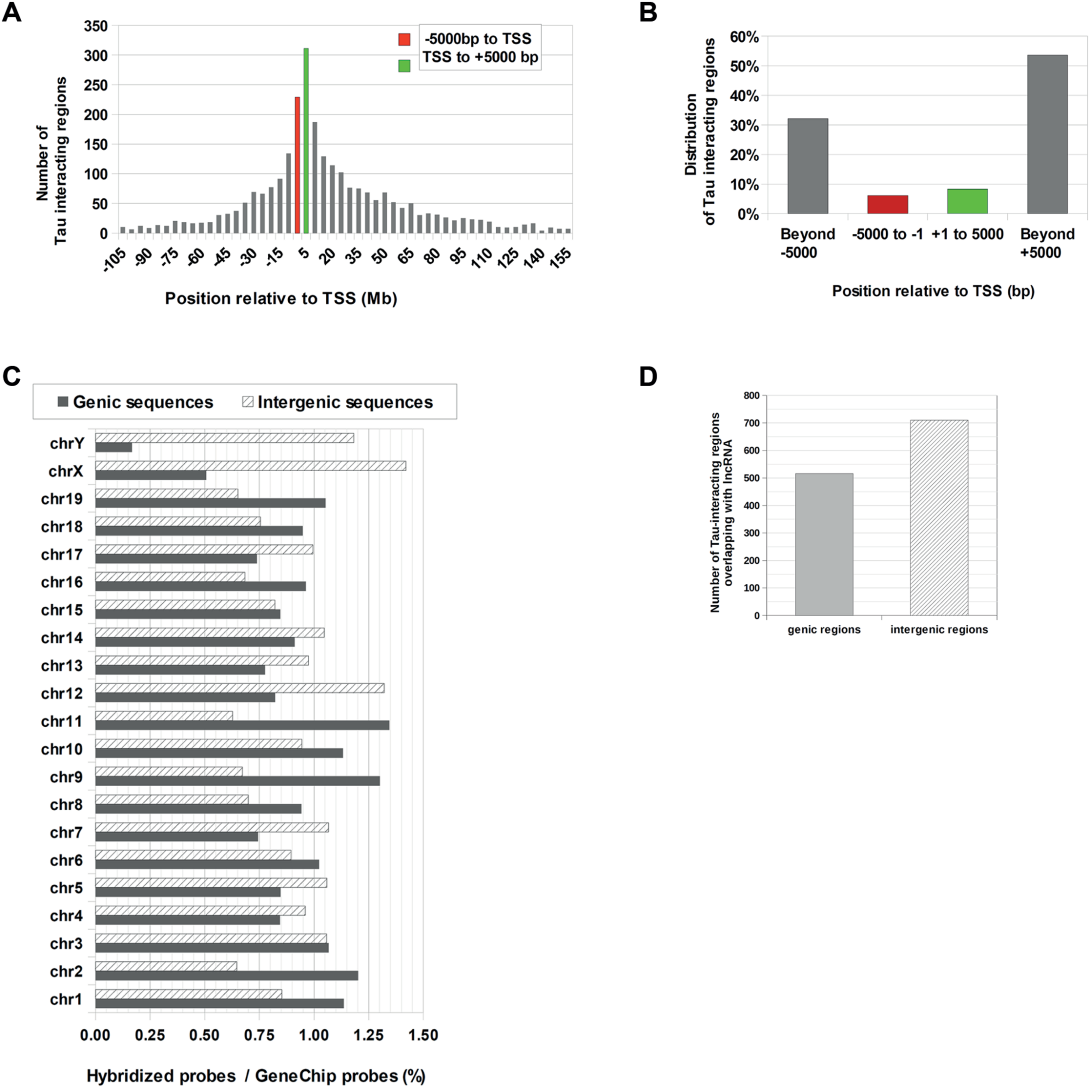
Tau protein preferentially interacts with DNA regions positioned beyond ±5000 bp from the TSS. Tau-interacting DNA regions were classified either as genic when positioned between 5000 bp upstream of TSS and 5000 bp downstream of STOP codon of an annotated gene or as intergenic when positioned outside this range. (**A**) Distribution of genic Tau-interacting regions according to their position upstream (–) or downstream (+) of TSS. (**B**) Percentage of genic Tau-interacting DNA regions positioned within ±5000 bp from TSS or beyond. (**C**) Distribution among chromosomes of genic and intergenic Tau-interacting probes expressed as a percentage of the total number of probes per chromosome present in the array. (**D**) Number of genic and intergenic Tau-interacting regions overlapping with DNA regions coding for lncRNA.

Results generated through PGS analysis were saved as text files (.txt) and further analyzed with a spreadsheet software. When interrogation of the RefSeq Transcripts database with Tau-interacting DNA regions had given rise to several transcript ID associated to a same Tau-interacting region, the region was considered as a single region (regardless the number of transcripts associated to it) while determining the number of Tau-interacting DNA regions, of genes associated to Tau-interacting regions (total and *per* chromosome) and of probes associated to Tau-interacting regions (*per* region and chromosome) (Figures 1C, 2, 3, 6, 7). Was taken into account the fact that a single Tau-interacting DNA region may overlap with more than one gene. Proportion of Tau-interacting probes and genes *per* chromosome (Figures 2A, 3C, 6C, 7A) was evaluated in reference to Affymetrix GeneChip parameters reporting the number of probes *per* chromosome present within the array. When evaluating the distribution of Tau-interacting regions relative to TSS (Figure 3A, B) and calculating mean transcript lengths (Figure 2D), only one transcript *per* region was considered for analysis. The overlap between Tau-interacting DNA regions and enhancers or LncRNA (Figure 1C and 6B) was tested by comparing their coordinates to those of the corresponding databases, VISTA Browser and NONCODEV5 respectively. Practically, this test determined if the start and/or end position of each Tau-interacting region was included between the coordinates of each enhancer or Lnc: ( = SI((Tau-interacting DNA region start position ≥ LncRNA start position) ET(Tau-interacting DNA region start position ≤ LncRNA end position)) then ( = SI((Tau-interacting DNA region end position ≥ LncRNA start position) ET(Tau-interacting DNA region end position ≤ LncRNA end position)); answer to the test would be ‘FALSE’ or ‘TRUE’. Eventual redundancies, occurring when Tau-interacting regions where entirely included into the enhancer/LncRNA sequence, were eliminated. Then, ‘true’ answers were summed for each Tau-interacting region.

### RNA collection from adult mouse brain

The brain was extracted after mouse decapitation. The cortex and hippocampus were dissected and flash frozen in liquid nitrogen. Samples were stored at −80°C. Total RNA of cortex and hippocampus from 17m KOTau and WT littermates, and hippocampal CA1 region from 6m THY-TAU22 and WT littermates were extracted using the RNeasy lipid tissue kit (QIAGEN, cat#74804) according to the manufacturer's recommendations. After elution, RNAs were treated with 1 unit/μl of DNAse I (Sigma) to remove genomic DNA. RNA concentration and purity (*A*_260_/*A*_280_) were determined using nanodrop.

### RT-qPCR

RT-qPCR assays were performed in conformation with MIQE guidelines (http://www.rdml.org/miqe.php). One μg of total RNA was reversely transcribed using High Capacity cDNA Reverse Transcription Kit (Applied Biosystems) according to the manufacturer's recommendations using Random Primers. qPCR assays were performed using a Biorad CFX384/qPCR Instrument, from 5 ng of the corresponding cDNAs in 10 μl final volume with SYBR Green (Bioline) reagents: 95°C 2 min, then 40 cycles at 95°C 5 s/60°C 10 s/72°C 15 s, followed by a dissociation step. Primers for qPCR analysis used in this work were manufactured by Eurofinsgenomics with the corresponding sequences listed in Supplementary Table S4. PCR efficiency was calculated from amplification slope for each couple of primers; control RT minus assays were carried out in order to verify specific amplification of cDNAs with respect to genomic DNA. Three technical replicates were performed for each qPCR assay. Relative quantification of mRNA expression was calculated using the Δ*C*_T_ method with respect to three reference genes (*Ppib, Hmbs, Rplp0*).

### *In vivo* hyperthermia model

The *in vivo* mouse model of transient hyperthermic stress was performed as previously described (12). Briefly, mice were anesthetized using xylazine (20 mg/kg) and ketamine (100 mg/kg) and maintained in a 37°C environment for 30 min. The mice were then maintained at 37°C (control (CTRL) group) or heat stressed (HS group) by being placed in an incubator containing ambient air heated to 44°C for 20 min. The rectal temperature of the mice was monitored every 10 min and did not exceed 41°C.

### Immunofluorescence

Immnufluorescence was performed as described previously (13). The TOC1 antibody (a gift from Dr Nicholas Kanaan, Michigan State University) that specifically targets prefibrillar Tau oligomers (32,33) was used as a primary antibody and the Alexa 488 (Life Technologies) as secondary antibody. The sections were counterstained and mounted with Vectashield/DAPI (Vector Laboratories). 4′,6-Diamidino-2-phenylindole (DAPI) was used as a chromatin counterstain. Nuclear TOC1 labeling (based on DAPI detection) of CA1 cells was quantified using the FIDJI macro application of ImageJ (confocal microscopy platform, IMPRT, Institut de Médecinr Prédictive et de Recherche Thérapeutique, Lille). A non-parametric Mann–Whitney U-test was used for statistical analysis of the quantification of TOC1 labeling.

### Ethics statement

All the animals were kept in standard animal cages under conventional laboratory conditions (12 h/12 h light/dark cycle, 22°C), with ad libitum access to food and water. Animal experiments were performed with the approval of the local ethics committee (agreement CEEA 062010R), in compliance with the standards for the care and use of laboratory animals, and the French and European Community rules.

### Statistical analysis

In Figures 8A–C and 9C–E, statistical analysis was carried out using GraphPad Software. A two-tailed, unpaired Student's *t*-test was used to statistically analyze the data, two by two for all conditions used. Before the application of the Student's *t*-test, the Shapiro–Wilk test of normality was used to verify that the data to be analyzed were normally distributed and the *F*-test of equality of variances was used to verify that variances were not significantly different. In Figure 8A–C, *n* = 17 (WT CTRL), 17 (WT HS), 13 (KO CTRL) and 17 (KO HS) animals. In Figure 9C–E, *n* = 11 (WT CTRL), 11 (WT HS), 12 (Tau22 CTRL) and 11 (Tau22 HS) animals.

## RESULTS

### Widespread distribution of Tau protein on chromatin under physiological condition

To identify neuronal DNA regions bound by Tau protein we performed genomewide ChIP-on-chip experiments that combine Chromatin ImmunoPrecipitation (ChIP) with DNA microarray (chip). Chromatin prepared from mouse primary cultures of embryonic cortical neurons was immunoprecipitated with Tau1 antibody. Tau1 recognizes Tau protein unphosphorylated at sites present between residues 189 and 207 and has been previously used to evidence Tau protein in the nuclear compartment (17,34,35) under physiological and heat stress (HS) conditions (11,12) as well as Tau protein interaction with DNA by ChIP (11,15). An antibody directed against the viral NSs protein encoded by Rift Valley Fever Virus (31) was used as a negative control. Neuronal DNA immunoprecipitated with Tau1 and anti-NSs antibodies were hybridized to the GenChIP Mouse Promoter 1.0R Array (Affimetrix) containing over 4.6 million Perfect Match probes designed to specifically interrogate over 26 000 mouse promoter regions. Probes in this array are tiled at an average resolution of 35 bp, as measured from the central position of adjacent 25-mer oligonucleotides, leaving a gap of ∼10 bp between probes (Figure 1A). Each promoter region covers ∼6 kb upstream through 2.5 kb downstream of 5′ transcription start sites. The data from three independent ChIP-on-chip experiments was analyzed by Partek Genomic Suite (PGS) that uses Model-based Analysis of Tiling-array (MAT) algorithm to detect DNA regions significantly enriched by ChIP-on-chip (36).

Instead of estimating probe behavior variations from multiple samples, MAT relies in its capacity to determine true biological enriched signals from baseline probe behavior. The MAT algorithm is a powerful 81 parameters probe behavior model that has been specially designed to analyze results from tiling-arrays that contain a very high number of probes giving rise to a large quantity of data as in the case of the ChIP-on-chip assays performed here. Because MAT does not rely on variations from multiple samples, it can handle a single ChIP-on-chip sample. However, its sensitivity and specificity is highly increased while analyzing multiple ChIP and control samples such as in this work, confidently detecting enriched ChIP regions with a reduced false discovery rate (36).

The different steps of the analysis of the data obtained from ChIP-on-chip assays carried out with Tau1 and anti-NSs (NSs) antibodies are summarized in Figure 1A. Firstly, Tau1 and NSs probe hybridization signals were determined. The original Affymetrix raw intensity files originated from the three independent Tau and NSs ChIP-on-chip assays were imported into PGS and processed with RMA (Robust Multi-array Average) background correction and quantile normalization algorithms. Secondly, variations among sample abundance of data from the three Tau1 and NSs biological replicates were determined by submitting RMA-processed fluorescence intensity data to a statistical Principal component analysis (PCA). Figure 1B shows the PCA plot of the three replicates of Tau1 (blue) and NSs (red) samples. Samples corresponding to the three Tau1 ChIP-on-chip assays clustered close together translating little variation among replicates. Whereas the samples resulting from the three NSs ChIP-on-chip assays displayed a scattered distribution, far away from each other, translating a high degree of variation among replicates as expected for random non-specific hybridizations. Thirdly, the log2 transformed RMA-processed fluorescence intensity data was subjected to a two-way ANOVA test. Fourthly, the probe fluorescence intensity data of each sample was MAT scored in order to determine true biological enriched signals. Finally, Tau-interacting DNA regions were determined. The conditions for considering a DNA region as a Tau-interacting region were: a minimum number of 10 contiguous probes with all probes from all samples displaying true positive (Tau1 being enriched with respect to NSs signals) biological enriched signals as determined by positive MAT scores. A maximum fraction of 0.1 of the total number of probes in the region was accepted as being potentially excluded from calculation. The fraction of negatively enriched windows in region is indicated in Supplementary Table S1.

The total number of probes present within DNA regions identified as significantly interacting with Tau compared to NSs under physiological (control) conditions corresponded to <2% (1.9%) of the total number of probes present in the array. The list of Tau-interacting DNA regions was used to interrogate the RefSeq transcripts database. Despite the fact that the array was designed to be enriched in promoter regions, DNA regions present outside the range of regions classified as promoter regions by the manufacturer were also found present among the DNA regions determined as significantly enriched in Tau1 vs anti-NSs ChIP-on-chip assays. Thus, Tau-interacting DNA regions were classified either as genic when positioned between 5000 bp upstream of TSS and 5000 bp downstream of STOP codon or as intergenic when positioned outside this range.

The general characteristics of the DNA regions determined as Tau-interacting regions under physiological conditions are described in Figure 1C. A similar number of Tau-interacting regions were classified as genic and intergenic. Interestingly, a significant amount (29.7%) of Tau-interacting regions overlapped with DNA regions coding for long non-coding RNAs (lncRNAs) whereas only a very low fraction of them overlapped with known enhancer regions (0.17%).

The Tau-interacting DNA probes were evenly distributed among all chromosomes (Figure 2A). The mean value of the percentage of Tau-interacting DNA probes compared to the total number of probes per chromosome present on the array were of 1.8% (±0.2 S.D.) for all chromosomes except the Y chromosome (1.35%).

Tau-interacting DNA probes clustered in 2138 genic DNA regions that overlapped with 1866 annotated genes coding for one or more transcripts (Supplementary Table S1). The vast majority of such Tau-interacting genes (83%) contained only one Tau-interacting region, ∼13% contained two regions, 2.5% three regions and 23 genes (1.2%) contained >3 and up to 12 distinct Tau-interacting regions (Figure 2B and C). Globally, the number of Tau-interacting regions in a gene was positively correlated with the corresponding transcripts length (Figure 2D). The mean length of the transcripts of the genes contacted by Tau at only one region was 100 000 bp, while the mean length of the transcripts of the genes contacted by Tau at four regions was 350 000 bp. The mean length of the transcripts of the gene containing the highest number of Tau-interacting regions, *Dlg2* (discs, large homolog 2, identified in Drosophila) was 1,97 megabasepairs (Mb) long.

The distribution of Tau-interacting genic DNA regions with respect to the TSS was analyzed (Figure 3). Tau-interacting genic DNA regions positioned around the TSS (±5000 bp) appeared over represented (Figure 3A). However, this could merely reflect the composition of the GeneChip array designed to cover from 6 kb upstream through 2.5 kb downstream of the TSS. Indeed, compared to the sum of Tau-interacting regions, the regions positioned around (±5000 bp) the TSS corresponded to only 13% of the total amount of Tau-interacting genic DNA regions (Figure 3B). The vast majority (87%) of the Tau-interacting genic DNA regions were positioned beyond the range of ±5000 bp from the TSS and this despite that probes positioned beyond ±5000 bp from TSS are expected to be under represented in the array.

Besides genic DNA regions, some 1989 Tau-interacting regions were classified as intergenic by PGS Software gene section analysis (Supplementary Table S2), a number comparable to that classified as genic (Figure 3C). On average, 0.92% of total Tau-interacting probes matched with genic sequences (positioned from −5000 bp, upstrean TSS, to +5000 bp downstream of STOP codon) and 0.92% probes matched with intergenic sequences. Tau-interacting regions that overlapped with DNA sequences coding for lncRNAs were present among genic as well as intergenic sequences with a preference toward intergenic sequences (Figure 3D).

Thus, overall ChIP-on-chip results obtained under physiological conditions showed that Tau protein recognized by Tau1 antibody specifically interacted with a fraction of neuronal chromatin with Tau-interacting regions being evenly distributed among all chromosomes as well as between genic and intergenic classified regions. Tau-interacting regions were mainly positioned further away than ±5000 bp from TSS with a preference towards DNA sequences coding for lncRNAs.

### An AG-rich DNA-binding motif was recurrently present within Tau-interacting regions

**Figure 4.**
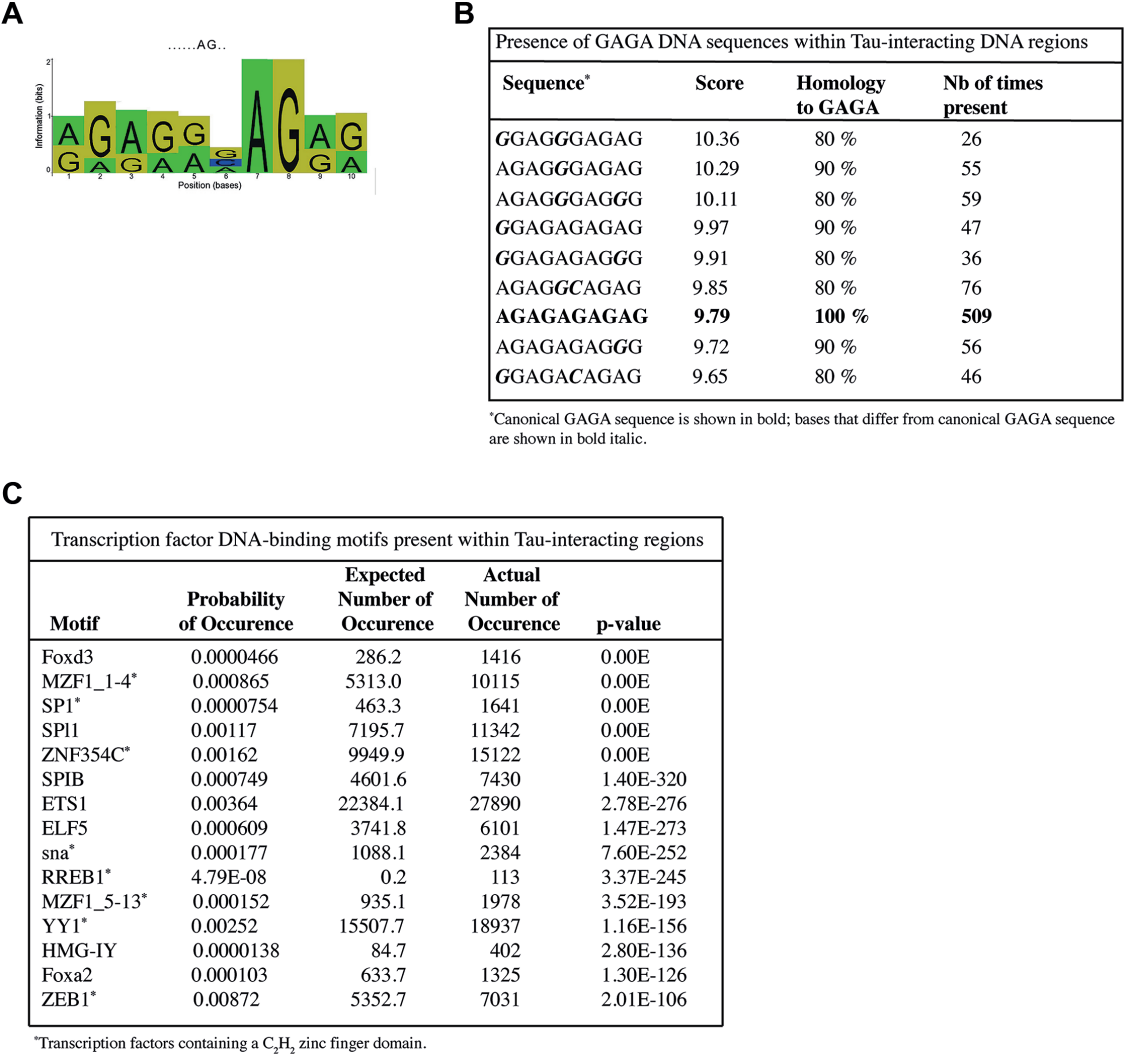
Determination of *de novo* and known DNA motifs present within Tau-interacting regions. (**A**) Sequence logo of the AG-rich *de novo* DNA motif identified as recurrently present within Tau-interacting regions. The height of each position indicates the importance of a base at a particular location in the binding site. The title, *……AG.*., defines the potential consensus sequence for the sequence logo. (**B**) Presence of the GAGA sequence among Tau-interacting regions. Nine out of the 15 highest scored sequences complying to the AG-rich *de novo* motif are shown. (**C**) List of the top 15 over-represented known DNA-binding motifs recognized by mammalian TFs present among Tau-interacting regions with, *Probability of Occurrence*: probability of detecting a false positive for this motif in a random DNA sequence; *Expected Number of Occurrence*: Probability of Occurrence multiple by the summed length of the reads; *Actual Number of Occurrence*: count of sequences that match the known motif in the reads.

A Tau-interacting DNA-binding motif was searched within Tau-interacting regions using the Gibbs motif sampler in PGS. A 10 bp *de novo* DNA-binding motif was identified, which was present 8010 times among the 4127 Tau-interacting regions (Figure 4A). Even though no clear consensus DNA sequence could be drawn from this motif, it displayed a specific pattern: over 90% purine rich with an almost exclusive AG composition. A total of 237 motif-variant sequences complied with the motif scoring from 10.36 to 7.2 (with scores corresponding to the log ratio of the probability that the sequence was generated by the motif versus the background distribution). On average, each variant sequence was present 34 ± 39 times among the whole set of Tau-interacting regions. However, among the motif-variant sequences, the variant 5′-AGAGAGAGAG-3′ was by far the most represented. It was present 509 times and corresponded to the highly conserved GAGA sequence known to regulate genome expression and stability through nuclear organization and chromatin remodeling (37,38). The GAGA sequence was present among the 15 highest scored variant sequences alongside with eight other variant sequences that differed from the GAGA sequence by no more than one or two nucleotides (Figure 4B).

In order to unveil potential Tau-interacting partners, the co-occurrence of Tau and known transcription factors (TFs) on the same DNA regions was searched using PGS through the JASPAR database. The list of the top 15 over-represented known motifs recognized by mammalian TFs is shown in Figure 4C. Eight (53%) out of the 15 TFs belonged to the family of TFs carrying a Zinc finger (ZF) C_2_H_2_ motif that correspond to 34% of the TFs expressed in the mouse brain (39). Transcription factors containing an ETS-domain (SPI1, SPIB, ETS1 and ELF5) or a Forkhead-domain (Foxd3 and Foxa2) also appeared over-represented since they constituted 27 and 13% respectively of TFs listed in Figure 4C while they have been identified as corresponding to only 2 and 2.7% respectively of TFs expressed in the mouse brain (39). On the contrary, homeobox- and bHLH-domain TFs appeared under-represented since even though they respectively constitute the second and third most abundant family of TFs expressed in the mouse brain (39), they were totally absent from the list of the top 15 over-represented known TFs motifs present in Tau-interacting regions (Figure 4C) that constitutes a first list of potential interacting partners for nuclear Tau protein.

### Neurological processes were significantly represented among Tau-interacting genes

**Figure 5.**
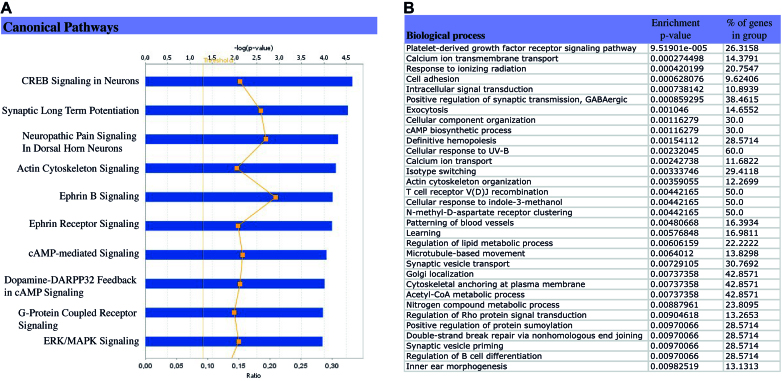
Neurological processes are significantly represented among Tau-interacting genes. IPA and PGS softwares were used to carry out the functional analysis of Tau-interacting genes under physiological conditions. (**A**) Top 10 pathways represented among the set of Tau-interacting genes as determined by IPA, ranged by their *P*-value (blue bars) as well as their ratio value (yellow squares) that corresponds to the percentage of Tau-interacting genes among the total number of genes in a given pathway. Threshold corresponds to *P*-value = 0.05. (**B**) Biological processes significantly over-represented (enrichment *P*-value <0.01) among Tau-interacting genes using PGS software.

In order to analyze the potential relevance of Tau interaction with the neuronal genome with respect to neurological functions and disorders, a functional analysis of Tau-interacting genes was carried out using Ingenuity Pathway Analysis (IPA) and PGS softwares. Interestingly, canonical pathways (Figure 5A) and biological processes (Figure 5B) identified as significantly enriched among Tau-interacting genes are, for the most, closely related to neurological functions and Tau-associated neurodegenerative disorders.

Among the ten most significant canonical pathways generated by IPA from the list of Tau-interacting genes (Figure 5A), the majority of the pathways were directly related with the biology of neurons and several of them (i.e. CREB signaling in neurons, Synaptic long-term potentiation, Ephrin signaling, c-AMP signaling, ERK/MAPK signaling) have been described as disturbed in AD (40–45) potentially linking Tau-interacting genes with tauopathies.

Further emphasizing the potential link between Tau-interacting genes and neurodegeneration, a majority of the biological processes identified by PGS as significantly enriched (*P*-value< 0.01) (Figure 5B) corresponded to neurological functions affected in neurological diseases such as AD. This is the case of biological processes such as learning and synaptic transmission as well as of PDGF receptor signaling (46,47), calcium transport (48) and c-AMP and NMDA receptor signaling (49). Interestingly, among biological processes significantly enriched among Tau-interacting genes were present processes related with DNA damage response (i.e.: response to ionizing radiation, cellular response to UV-B, double-strand break repair via nonhomologous end joining) potentially linking, in this case, Tau-interacting genes with the DNA protective function of Tau protein (11–13).

### Heat stress induced a global dissociation and redistribution of Tau protein on chromatin

**Figure 6.**
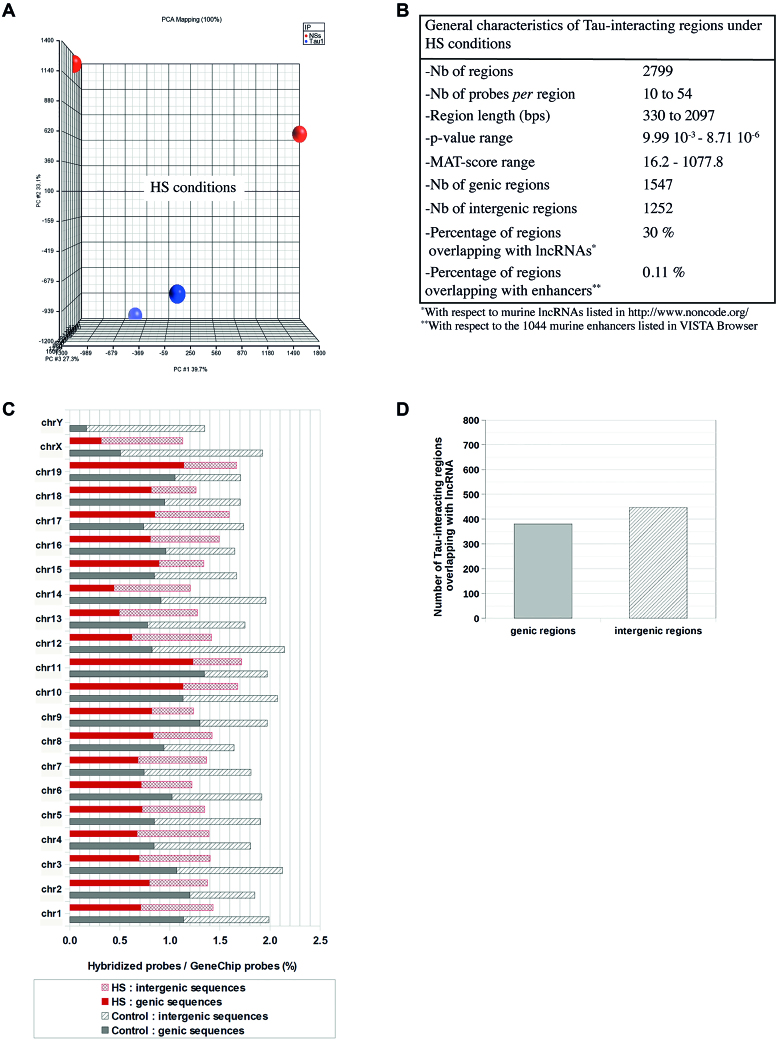
General characteristics of Tau-interacting regions identified under HS conditions. General characteristics of the DNA sequences identified through ChIP-on-chip technique, Tau1 versus anti-NSs (NSs) antibody, as Tau-interacting regions under HS conditions. (**A**) Principal component analysis (PCA) plot that separates samples according to abundance variation showing that under HS conditions Tau1 samples cluster tightly (little variation) compared to non-specific anti-NSs (NSs) samples that displayed strong variation. (**B**) General characteristics of DNA regions identified as significantly enriched in Tau1 samples under HS conditions, considered as Tau-interacting regions. The *P*-value of the region corresponds to the empirical *P*-value (as determined using a two-way ANOVA test) of the most significant probe MAT score included within this region and the MAT score of the region is the maximum probe MAT score for this region. The *P*-values and MAT scores of Tau-interacting regions are indicated in Supplementary Table S3. (**C**) Distribution among the chromosomes of the DNA probes bound by Tau protein, either genic or intergenic, under physiological (control) and HS conditions expressed as a percentage of the total number of probes per chromosome present in the array. **D**) Number of genic and intergenic Tau-interacting regions overlapping with DNA regions coding for lncRNA under HS conditions.

In order to investigate the plasticity of Tau interaction with DNA, we analyzed the effect of HS on the capacity of Tau protein to interact with neuronal DNA. The choice of HS was justified by results obtained in previous works showing that HS affected neuronal Tau phosphorylation, and subcellular distribution by enhancing the presence of Tau protein in the nuclear compartment (11–13). In order to determine the effect of HS on the capacity of Tau protein to specifically interact genomewide with neuronal DNA sequences, three independent mouse primary cultures of embryonic cortical neurons were submitted to HS previous to ChIP-on-chip assays with Tau1 and anti-NSs antibodies. DNA immunoprecipitated with Tau1 and anti-NSs antibodies were hybridized to the GenChIP Mouse Promoter 1.0R Array (Affimetrix) and analyzed alongside with immunoprecipitated DNA from cultures carried out under physiological conditions as described previously. Only two out of the three Tau1 biological replicates conformed the control quality test. The corresponding PCA plot show that, as under physiological conditions, the Tau1 samples were close to each other (Figure 6A) translating little variation among replicates compared to the NSs HS samples that displayed a scattered distribution, far away from each other, translating a high degree of variation as expected for random non-specific hybridizations.

DNA fragments immunoprecipitated with Tau1 under HS condition hybridized with 1.4% of the array total probes, that is 25% less than under physiological conditions suggesting a trend towards Tau DNA dissociation after HS notwithstanding the HS-induced nuclear accumulation of Tau isoform recognized by Tau1 antibody (11). The general characteristics of the 2799 DNA regions identified as Tau-interacting regions under HS conditions are described in Figure 6B.

As observed under physiological conditions, Tau-interacting probes under HS conditions were also evenly distributed among chromosomes and between genic (0.77%) and intergenic (0.63%) regions (Figure 6B and C). They clustered in 1547 genic DNA regions (listed in Supplementary Table S3) that overlapped with 1353 annotated genes that is 30% less than under physiological conditions. As under physiological conditions, a significant amount (30%) of the HS Tau-interacting regions overlapped with DNA regions coding for lncRNAs whereas only a small fraction of them overlapped with known enhancer regions (0.11%) (Figure 6B). HS Tau-interacting regions overlapping with DNA sequences coding for lncRNAs were evenly distributed among genic and intergenic DNA regions (Figure 6D).

**Figure 7.**
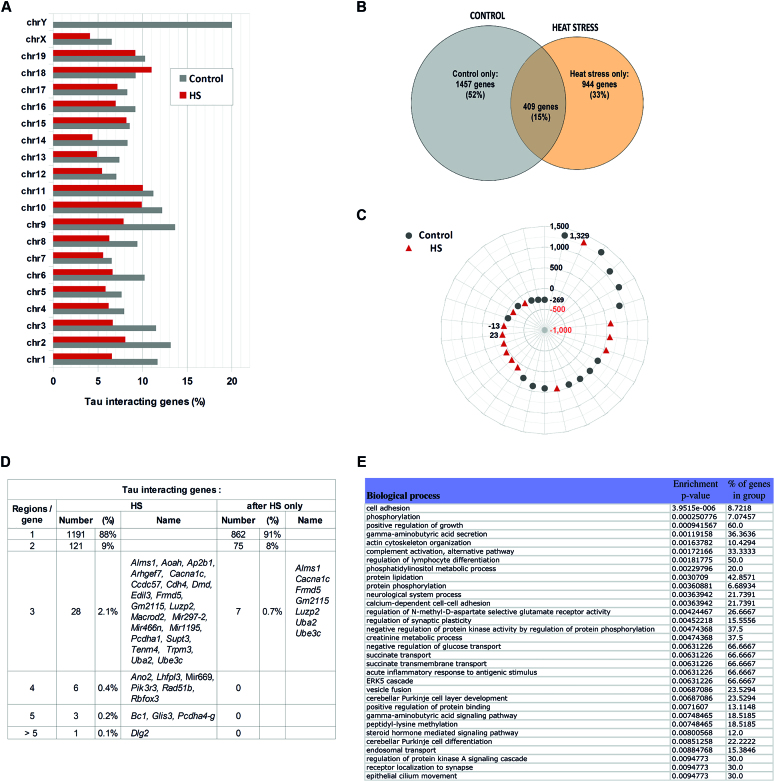
Heat stress induces a dissociation and re-localisation of Tau protein on neuronal chromatin. (**A**) Distribution among the chromosomes of Tau-interacting genes expressed as a percentage of the total number of genes bound by Tau protein either under control or HS conditions. (**B**) Number of genes bound by Tau protein either under physiological and HS conditions or only under physiological (control) or HS condition. (**C**) Distribution of Tau-interacting regions along the *Dlg2* gene as determined under physiological (control) and HS conditions; regions are positioned relative to *Dlg2* TSS (kb). (**D**) Number of genes displaying 1 to >5 Tau-interacting regions and list of those displaying three or more Tau-interacting DNA regions. (**E**) Biological processes significantly over-represented (enrichment *P*-value < 0.01) among Tau-interacting genes under HS conditions, as determined using PGS software.

HS-treatment differentially affected Tau interaction with genes present on different chromosomes. In the case of several chromosomes (e.g. 4, 5, 7, 11, 15, 17, 19) the percentage of Tau-interacting genes was only slightly diminished after HS compared to physiological conditions whereas it was enhanced in the case of chromosome 18 and divided by almost two in the case of chromosomes 1 and 14 (Figure 7A).

Alongside with a global trend towards dissociation from chromatin, a relocalization of Tau protein within chromatin was observed so that Tau protein interacted with a different set of genes in response to HS as compared to physiological conditions (Figure 7B). Comparison of the list of Tau-interacting genes under physiological and HS conditions revealed that among the 1353 genes bound by Tau under HS, 944 genes corresponded to new genes bound by Tau only after HS. Genes bound by Tau under both control and HS conditions (listed in Supplementary Table S5) represented only 15% of the overall genes bound by Tau (Figure 7B). Besides, in this subset of genes, Tau interacted mainly within distinct regions, as sketched for example in the case of the *Dlg2* gene (Figure 7C). Not only the number of Tau-interacting genes decreased after HS but, globally, also diminished the proportion of genes containing several Tau-interacting regions (Figure 7D). Of the genes bound by Tau only after HS, 91% contained only one Tau-interacting region and none contained more than 3 regions.

In agreement with changes in Tau occupancy, the biological processes identified by PGS as significantly enriched (*P*-value < 0.01) among Tau-interacting genes after HS (Figure 7E) differed from those identified under physiological conditions. Neurological functions were still represented, however these functions appeared related to the cerebellum (mainly Purkinje cells), thus mainly implicated in motor coordination and to a lesser extent in some cognitive functions, such as attention and language, rather than learning and memory as under physiological conditions. Also, functions such as DNA repair and calcium transport that were significantly represented among Tau-interacting regions under physiological conditions were no longer represented after HS. Whereas, protein phosphorylation and inflammatory response that were absent under physiological conditions were found represented after HS.

### A transcriptional repressor role for Tau protein on genes associated to Tau-interacting regions

In order to investigate the capacity of Tau protein to affect the expression of genes associated to Tau-interacting regions, a first set of gene expression assays was carried out using murine primary neuronal cultures of embryonic origin. For this, the expression of 30 genes among those identified as associated to Tau-interacting regions under physiological conditions was tested by RT-qPCR after purification of total RNA from embryonic cortical neurons from wild type (WT, three independent cultures) and Tau-deficient (KOTau, five independent cultures) mice. The 30 genes tested were selected according to their roles in biological processes associated to Tau protein and AD pathology (*Mark1, Aatf, Pkn, Anp32a, Mapk1*), neuronal networks and brain function (*Grm5, Cacna1a, Ntrk2a, Ppp2r2b, Atxn1, Ctnn1*), DNA Damage Response (*Ercc3, Ten1, Xrcc4, Ctc1*), transcription regulation and chromatin remodelling (*Suv39h2, Suv420h1, Jarid2, Creb1, Cbfa2t2, Ppargc1a, Cnot4, Phf21a, Smarca1, Mtf2, Polr2a*) or related with RNA-mediated gene silencing (*Ddx6, Tnrc6b*). Among the 30 genes selected, four were previously identified using functional genomic screening in *Drosophila* as modifiers of tauopathy: *Mark1, Camk1, Ercc4* (50) and *Nab1* (51) and three were previously identified during microarray assays as deregulated in KOTau versus WT primary neuronal cultures (MC Galas, unpublished results): *Cbfa2t2, Cnot4, Tnrc6b*.

The transcription rates of the 30 genes in WT and KOTau neurons were calculated with respect to 3 reference genes (*Ppib, Rplp0* and *Hmbs*), absent from the list of Tau-interacting genes and displaying similar expression rates in WT and KOTau neurons (data not shown). Among the 30 Tau-interacting genes tested, seven genes displayed over a 1.5 fold change in WT versus KOTau neurons with their respective transcriptional rates enhanced in KOTau compared to WT neurons: *Grm5* (2.25 ± 1.25), *Camk1* (1.68 ± 0.87), *Jarid2* (1.61 ± 0.87), *Ntrk2* (1.78 ± 0.99), *Ctc1* (1.58 ± 1.45), *Ctnn1b* (1.877 ± 1.52) and *Polr2a* (2.56 ± 1.98). However, because of strong variations among independent cultures (mainly of WT origin), these differences were not statistically significant (data not shown). In order to further explore the potential role of Tau protein regulating the expression of Tau-interacting genes, Tau-dependent gene expression analysis was extended to *in vivo*, physiological versus HS conditions.

**Figure 8.**
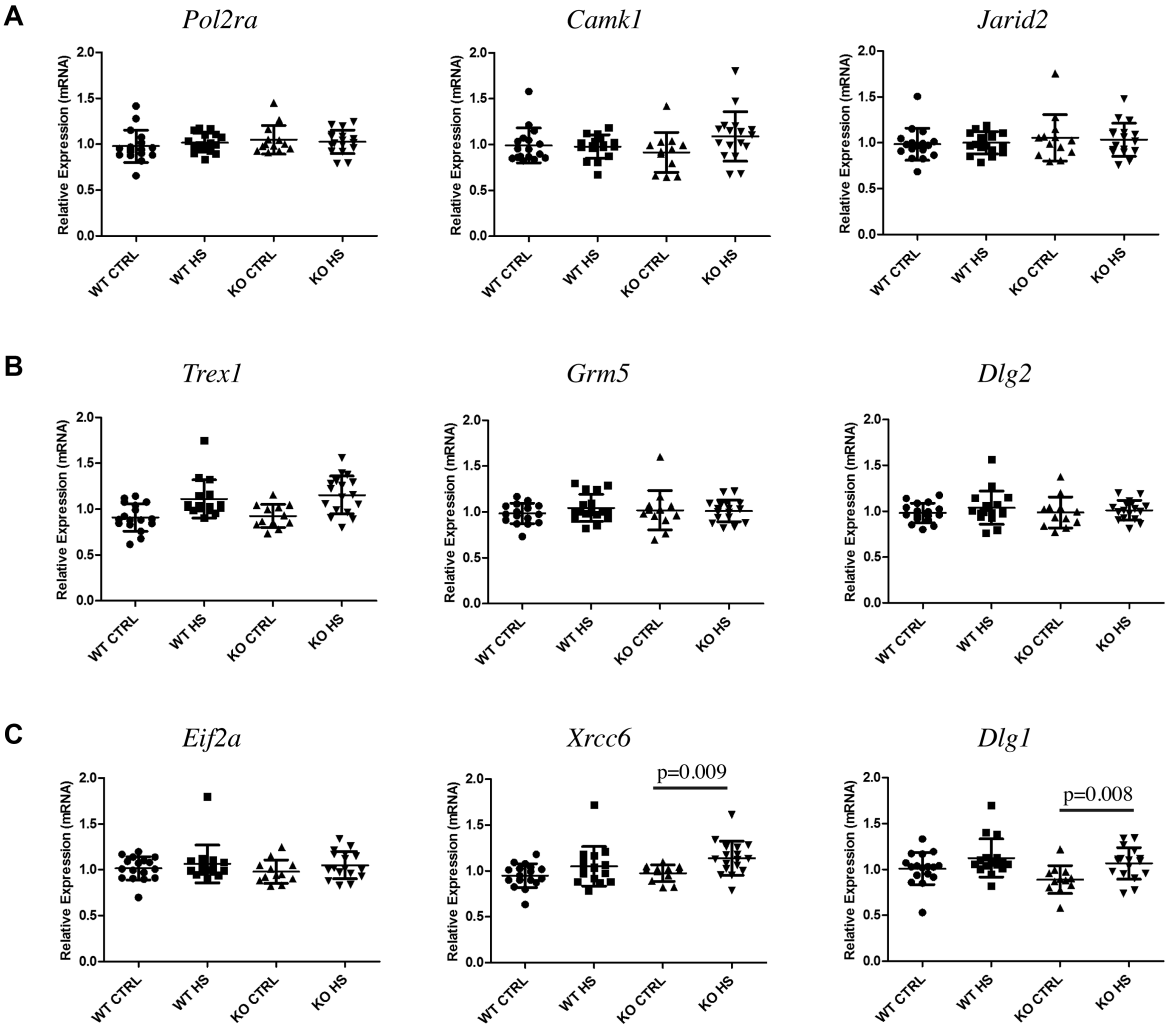
The absence of Tau protein enhanced the expression of Tau-interacting genes after HS treatment. Relative gene expression was analyzed by RT-qPCR with respect to three reference genes (*Ppib, Rplp0, Hmbs*). Total RNA was collected from brain extracts of 17-month-old mice either wild-type (WT) or deficient for Tau protein (KOTau), not submitted (CTRL) or after being submitted to HS (HS). Relative expression rates of mRNAs of genes interacting with Tau (**A**) only under physiological, (**B**) under physiological and HS conditions and (**C**) only under HS conditions are shown. In the graphs, each point corresponds to a unique animal. Quantification was carried out for n (number of mice) = 17 (WT CTRL), 17 (WT HS), 13 (KO CTRL) and 17 (KO HS). Data are means ± S.D. with *P*-values < 0.01 indicated.

In a second set of experiments, the investigation of the potential role of Tau protein in regulating the expression of Tau-interacting genes was extended to brain extracts from old WT and KOTau mice either not submitted (control, CTRL) or submitted to HS (Figure 8). For this, RNA was collected from either cortex or hippocampus extracts of 17-month-old mice either WT, not submitted (17 WT CTRL mice) and after being submitted to HS (17 WT HS mice), or KOTau not submitted (13 KO CTRL mice) and after being submitted to HS (17 KO HS mice). For each group of mice, we measured the expression rate of a set of three genes identified as bound by Tau either only under physiological conditions (Figure 8A), under physiological and HS conditions (Figure 8B) or only under HS conditions (Figure 8C).

Among the nine genes tested, four (*Polr2a, Camk1, Jarid2, Grm5*) were chosen from the previous group of seven genes bound by Tau under physiological conditions that displayed over 1.5-fold difference in KOTau versus WT primary neuronal cultures of embryonic origin. The other five genes, bound by Tau under HS conditions (as also *Grm5*), were chosen according to their implication in neurodegenerative diseases (*Trex1, Dlg2, Dlg1, Xrcc6, Eif2a*). Expression rates were calculated with respect to the same set of three reference genes than before. Of note, the RNAs extracted from cortex and hippocampus used in theses assays, originated not only from neurons that are Tau-expressing cells but also from glial cells that are poorly- or non-Tau expressing cells causing the attenuation of potential Tau-dependent effects.

No statistically significant effects were observed for the set of genes that interacted with Tau only under CTRL (Figure 8A) or under CTRL and HS (Figure 8B) conditions. Whereas, significant (*P*-value < 0.01) Tau-dependent gene expression variations were observed for two out of the set of three genes that interacted with Tau only under HS conditions (*Xrcc6* and *Dlg1*) (Figure 8C). For both of these genes, their expression rates were significantly enhanced in KOTau neurons after HS treatment as compared to CTRL conditions whereas they were not affected by HS in the case of WT neurons.

The significant gene expression variations observed here in relation with the absence of Tau protein suggest a transcriptional repressive role for Tau protein necessary in regulating gene expression under stress conditions. Gene expression changes have been described to rapidly occur after HS, affecting many biological pathways including mitochondrial dysfunction, altered protein synthesis and the immune response (52), which are biological processes also affected in association to tauopathies.

### The repressive role of Tau protein in regulating the expression of Tau-interacting genes was exacerbated in the presence of pathological prefibrillar Tau oligomers

**Figure 9.**
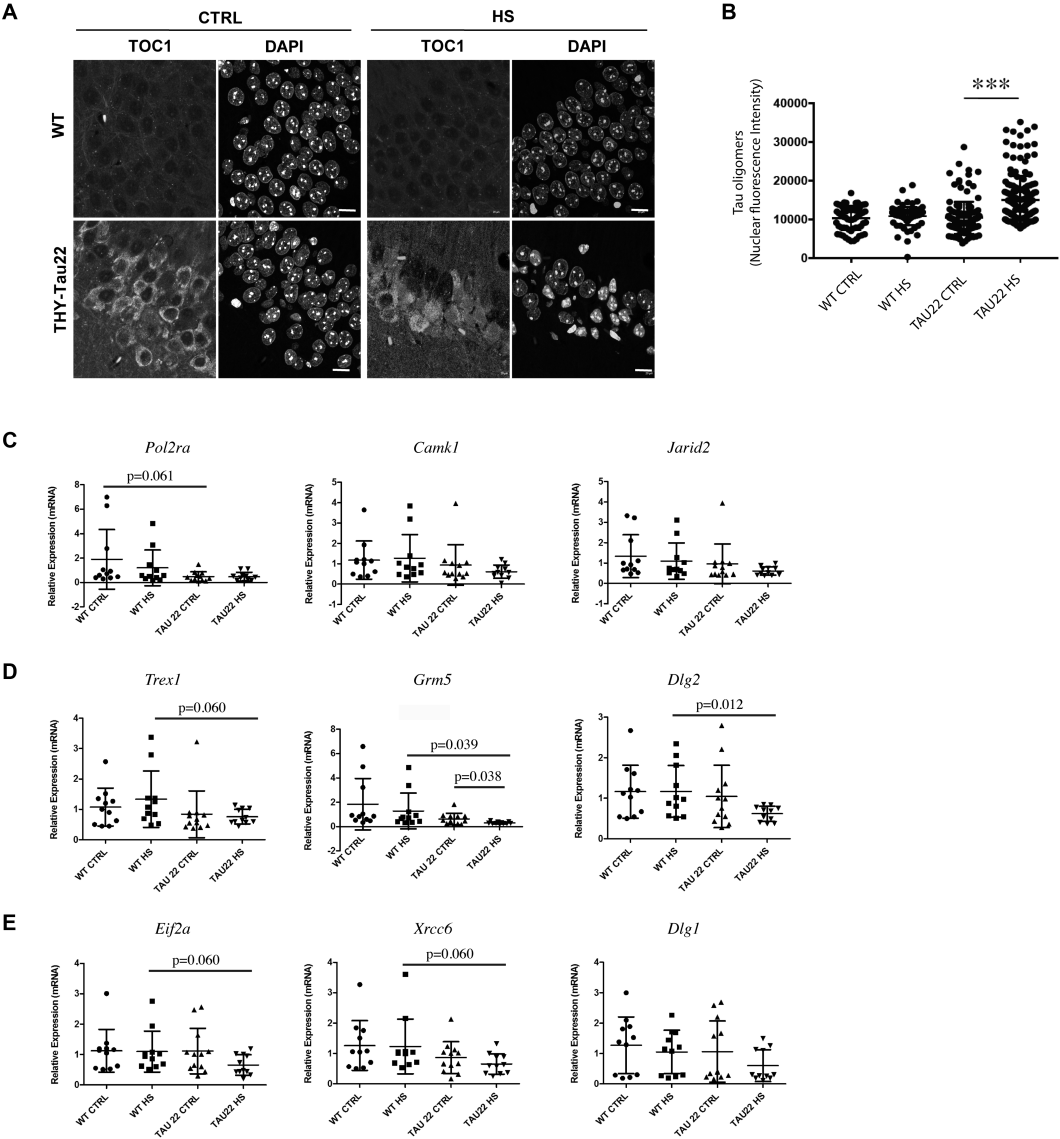
The presence of pathological prefibrillar oligomerized forms of Tau protein repressed the expression of Tau-interacting genes. Relative gene expression was analyzed by RT-qPCR with respect to three reference genes (*Ppib, Rplp0, Hmbs*). Total RNA was collected from the CA1 region of 6 month old mice either wild-type (WT) or THY-Tau22 expressing Tau oligomers, not submitted (CTRL) or after being submitted to HS (HS). (**A**) Representative images of sagital CA1 sections from 6-month-old WT and THY-Tau22 mice under CTRL and HS conditions labeled with the TOC1 antibody directed against prefibrillar Tau oligomers (32,33) and analyzed by laser scanning confocal microscopy. The nuclei were identified using DAPI staining. The scale bars represent 20 μm. (**B**) The labeling intensity of Tau-oligomers was quantified within the nuclei (based on DAPI detection) of CA1 cells. The data shown are the means ± s.d of 60 to 160 nuclei. ***P-values < 0.001. (C, D) RT-qPCR relative expression rates of mRNAs of genes interacting with Tau under: only physiological (**C**), physiological and HS (**D**) and only HS (**E**) conditions. In the graphs each point corresponds to a single animal. Quantifications were carried out for *n* (number of mice) = 11 (WT CTRL), 11 (WT HS), 12 (Tau22 CTRL) and 11 (Tau22 HS). Data are means ± S.D. with *P*-values ≤ 0.06 indicated.

In order to analyze the potential effect of Tau pathology on the expression of Tau-interacting genes and compare this effect to that induced by the absence of Tau protein, the expression rates of the nine Tau-interacting genes previously analyzed in brain extracts from WT and KOTau mice were further analyzed in hippocampal CA1 extracts of 6-month (6m) old THY-Tau22 mice with respect to CA1 extracts from WT littermates. The CA1 regions from 6m THY-Tau22 brains are characterized by the presence of pathological prefibrillar oligomerized forms of Tau protein as revealed by immunohistochemical analysis and confocal microscopy using the TOC1 antibody (13) (Figure 9A, B). Prefibrillar Tau oligomers that were mainly visible in the cytoplasm of THY-Tau22 CA1 neurons under CTRL conditions were observed, not only in the cytoplasm, but also accumulated in the nucleus of neurons after HS treatment (Figure 9A, B). In order to analyze the effect of pathological oligomerized Tau protein on Tau-interacting gene expression, RNAs were collected from dissected CA1 regions from 6m mice either WT, not submitted (11 WT CTRL mice) and after being submitted to HS (11 WT HS mice), or THY-Tau22 not submitted (12 Tau22 CTRL mice) and after being submitted to HS (11 Tau22 HS mice).

The differences on gene expression observed between THY-Tau22 and WT mice (Figure 9C–E) were more pronounced than those previously observed between KOTau and WT mice (Figure 8). A strong gene expression heterogeneity was observed in extracts from WT animals under CTRL and HS conditions for all the genes tested that was strongly attenuated in extracts from THY-Tau22 mice, specially under HS conditions (Figure 9C–E). Alongside with a diminution of gene expression heterogeneity, a decrease of gene expression was observed in THY-Tau22 extracts with respect to WT extracts, mainly under HS conditions, for all the genes tested (Figure 9C–E).

Notwithstanding the strong heterogeneity observed in WT animals, changes in gene expression rates were found statistically significant (*P*-values ≤ 0.06) in the case of 6 (*Pol2ra, Trex1, Grm5, Dlg2, Eif2a, Xrcc6*) out of the nine genes tested. For all genes, except *Polr2a*, gene expression variations were observed under HS conditions that corresponded to conditions of increased accumulation of oligomerized Tau in the nucleus of neurons (Figure 9A, B).

Overall, results obtained with CA1 extracts from THY-Tau22 mice indicated that Tau pathology affected the expression of Tau-interacting genes, specially after HS treatment under conditions of nuclear accumulation of oligomerized Tau. Contrary to the activation of gene expression observed in KOTau extracts, the presence of pathological forms of Tau appeared associated to the repression of gene expression, translating a gain of pathological function rather than a loss of physiological function of Tau protein.

## DISCUSSION

This work constitutes the first large scale genomewide analysis of Tau interaction with neuronal DNA. In agreement with data previously published by our group and others reporting the presence of Tau protein in the nucleus of neurons (11,12,18) as well as the capacity of Tau protein to form protein-DNA complexes (11,17,19–21), results obtained in this work demonstrate that Tau protein specifically interacts with a fraction of genic and intergenic DNA sequences of the genome of murine neurons.

In order to specifically question the capacity of Tau protein to interact with regulatory DNA regions positioned at the vicinity of the TSS, we used for ChIP-on-chip experiments an array enriched in sequences positioned near the TSS (Affymetrix Mouse Promoter 1.0R array). However, contrary to our expectations, results obtained from ChIP-on-chip assays clearly indicated that DNA regions bound by Tau were predominantly positioned outside ‘conventional’ promoter regions, that is beyond the ±5000 bp range from TSS. Tau-interacting DNA regions were evenly distributed among all chromosomes, for the most absent from ‘conventional’ promoter regions with half of the Tau-interacting DNA sequences positioned within non-protein coding intergenic regions. Thus, overall Tau protein displayed DNA-binding characteristics different from those expected for a conventional TF.

Thirty percent of Tau-interacting regions overlapped with DNA regions coding for lncRNAs. LncRNAs are for the most enriched in the nuclear compartment where they participate in the regulation of transcription and translation as well as in genome organization (53). A potential role for Tau protein in genome spatial organization would be in line with defects in genome organization that have been observed in the context of Tau deficiency and pathology (14,15,18) and of interest in face of recent studies that point out the contribution of genome organization and 3D structure of chromosomes in neuronal function and plasticity (54), including a role for deregulation of genome organization in relation with aging and AD (27).

Besides, many studies have identified lncRNAs associated to AD with lncRNAs exhibiting aberrant expression rates in AD (55). An antisense lncRNA to *Dlg2* gene overlaps the *Dlg2* gene, regulating its expression. Interestingly, the expression rate of the *Dlg2* gene, which is the genic DNA sequence displaying the highest number of Tau-interacting regions, was significantly deregulated in neurons from the CA1 region of THYTau22 mice under conditions of nuclear accumulation of pathological oligomerized forms of Tau protein. The deregulation of the expression of the *Dlg2* gene that codes for a key scaffolding protein enriched at postsynaptic membranes has been linked to AD (56). Future work should allow to characterize the role of Tau interaction with lncRNA annotated DNA sequences, which have been identified here as privileged Tau-DNA interacting regions, in the development of tauopathies.

The identification of the recurrent presence of an AG-rich GAGA-like DNA motif within Tau-interacting regions further supports a potential role for Tau protein in nuclear organization. Indeed, repeated AG-rich GAGA motifs that are evenly dispersed throughout the genome (as it was also the case for Tau-interacting regions) regulate genome 3D structure through boundary and loop organization (37). In mammalian cells, GAGA motifs are present in DNA lamina-associated domains regulating genome compartmentalization and chromatin silencing through the association of inactive chromosomal domains to the inner nuclear lamina (38). In *Drosophila* (57,58) and in plants (59), GAGA factors (GAFs) associate to and regulate heterochromatin structures. Interestingly, lamina dysfunction (60) and heterochromatin disruption (14,15) are phenotypes that have been associated to Tau-mediated pathology. Thus, it is tempting to hypothesized that modifications of Tau interaction with AG-rich GAGA motifs could be playing an active role in tauopathies.

After HS, Tau protein was found displaced to new sites of interaction so that the majority of Tau-interacting DNA regions identified after HS differed from those identified under physiological, non-HS conditions, indicative of a certain degree of plasticity in Tau genome occupancy. This plasticity appeared necessary to protect Tau-interacting genes from HS-induced gene-expression deregulation. Of note, in *Drosophila*, GAFs have been shown to play a critical role in regulating HS-induced gene expression (61), reinforcing similarities between Tau and GAFs.

A fraction of Tau-interacting regions were found associated to genic, protein-coding DNA sequences with the annotated genes associated with these regions being functionally related to several neurological processes affected during tauopathies. Notwithstanding the attenuation due to RNAs from cells not expressing Tau protein present in the extracts, a gene expression repressor effect on Tau-interacting genes was observed *in vivo*, in brain extracts from KOTau and THY-Tau22 mice, the latest ones expressing Tau oligomers that are considered as the most deleterious forms of Tau protein rather than aggregates, with respect to their corresponding WT littermates. The repressor role depicted for pathological oligomerized Tau protein upon Tau-interacting genes was predominantly observed under HS conditions in correlation with the increased presence of Tau oligomers within the nuclear compartment of neurons associating the presence of nuclear pathological oligomerized Tau with the repression of Tau-interacting genes.

Results achieved in this work were obtained using the Tau1 antibody that recognizes Tau protein unphosphorylated at sites present between residues 189 and 207 which has traditionally been used to evidence Tau protein in the nuclear compartment of neuronal and non-neuronal cells (11,12,17,34,35,62) under physiological and HS conditions (11,12). However, although Tau1 was the most relevant antibody to use in this context, Tau isforms other than those recognized by Tau1 have also been observed in the nucleus of neurons (18,63) and could display patterns of genome occupancy different from those determined here using the Tau1 antibody, such as oligomerized forms of Tau recognized by the TOC1 antibody as well as Tau phosphorylated at residues 212 and 214 and recognized by the AT100 antibody, whose nuclear accumulation varies with age and the AD-related Tau pathological state of neurons (18,63).

Although limited to Tau protein unphosphorylated between residues 189 and 207 recognized by the Tau1 antibody, results obtained here constitute a strong basis from which to go further on the analysis of Tau interaction with neuronal genomes. We believe that based on results obtained in this work, large-scale analysis of the capacity of Tau protein to interact with specific DNA regions of neurons will be extended to other forms of Tau protein as well as to diverse states of neurons from normal to pathological. Since Tau protein was identified here as interacting with non-protein coding regions with a preference for dispersed, repeated DNA motifs (such as the GAGA motif), future work should be carried out using techniques such as chromatin immunoprecipitation sequencing (ChIP-Seq) that allows for an unbiased exploration of the whole genome including repeated non-protein coding DNA sequences such as satellite, LINEs, SINEs, rDNA, telomere, etc. that were absent from the array used in this work. Recently, the activation of transposable elements has been associated to Tau pathology in *Drosophila* models of tauopathies and in AD brain (64), reinforcing a link between Tau protein and non-protein coding DNA sequences.

Beyond Tau protein, it would be of interest to extend this approach to other main actors of neurodegenerative diseases for which neuronal nuclear functions have also been postulated such as amyloid-β peptides and α-synuclein (65,66).

## DATA AVAILABILITY

The list of: (i) the genic DNA regions interacting with Tau protein under physiological conditions (Supplementary Table S1); (ii) the gene section classification of all DNA regions interacting with Tau protein under physiological conditions (Supplementary Table S2) and (iii) the list of genic DNA regions interacting with Tau protein under HS conditions (Supplementary Table S3) are available on line at http://microarrays.curie.fr/publications/UMRS1007/Benhelli-Mokrani_2018/.

## Supplementary Material

Supplementary Data
