# Supplementary material for: Genome-wide identification of genic and intergenic neuronal DNA regions bound by Tau protein under physiological and stress conditions
Source: Nucleic Acids Res. 2018 Oct 13;46(21):11405–22. doi: 10.1093/nar/gky929 (PMC6265482; doi:10.1093/nar/gky929)
Supplement: Supplementary Data [file gky929_supplemental_files.zip › Supplementary files NAR-02473-V-2018.R1 (amended).pdf]

Table S1. Sequences (5' to 3') of the different primers used in qPCR assays.

	Forward	Reverse
Polr2a	GCCAGGACACTCGGTCATG	TGCGCACCATCAAGAGAGTG
Camk1	CAAGAGCAAGTGGAAGCAAG	AGAGGTGTGGGGTCAGAAAC
Jarid2	GAAGGCGGTAAATGGGCTTCT	TCGTTGCTAGTAGAGGACACTT
Trex1	CAGCATCTGTCAGTGGAAGCC	GCCAGGGGTGTAGTAGC
Grm5	CCCAGCACAAGTCGGAAATAG	TGTCTGGTTGGGGTTCTCCTT
Dlg2	CTGTCACGAGGCAGGAAATAAA	CGACTTCGTAGTCACGCTTTG
Eif2a	CACCGCTGTTGACAGTCAGAG	GCAAACAATGTCCCATCCTTACT
Xrcc6	ATGTCAGAGTGGGAGTCCTAC	TCGCTGCTTATGATCTTACTGGT
Dlg1	CGAAGAGTCACGTCGTTTTGA	TCTCCAAAGCGGAAGTTCAGT
Hmbs	TCCCTGAAGGATGTGCCTA	AAGGGTTTTCCCGTTTGC
Ppib	GGAGATGGCACAGGAGGAAA	CCGTAGTGCTTCAGTTTGAAGTTCT
Rplp0	CACTGGTCTAGGACCCGAGAAG	GGTGCCTCTGGAGATTTTCG

Table S2 - List of genes interacting with Tau under Ctrl and HS conditions

Gene Symbol
1700024P16Rik, 1700113A16Rik, 2810004N23Rik, 4930443G12Rik, 4932438A13Rik, 4933406I18Rik, 4933409K07Rik, 4933426M11Rik, 9130019P16Rik, 9130206I24Rik, 9530036O11Rik, A330093E20Rik, A430089I19Rik, Aatk, Abca14, Abca3, Abcc5, Acaa2, Acly, Acpl2, Acss3, Actn1, Adcy4, Adcy9, Agbl1, Agbl4, Ahcy, Akap6, Alk, Alox12, Amtn, Ank3, Ankhd1, Anks1b, Ano2, Aoah, Ap2b1, Apc2, Aph1a, Arhgef28, Arhgef3, Arhgef7, Armc9, Asf1b, Asic2, Asrgl1, Atg10, Atg7, Atp11c, Atp2b2, Atrip, Auts2, BC028528, BC049762, Bahcc1, Bc1, Bcl11b, Bcl9, Bcl9l, Bicc1, Bmp8b, C230081A13Rik, C730002L08Rik, Cabin1, Cacna1a, Cacna1d, Cacna2d3, Cacnb4, Cald1, Calu, Catsper4, Cbl,Ccbe1, Ccdc13, Ccdc141, Ccdc151, Ccdc37, Ccdc46, Ccdc57, Ccdc84, Ccny, Cd300lh, Cdc37l1, Cdh13, Cdh4, Celf1, Celf4, Cerk, Cerkl, Chd7, Chn1, Chn2, Cinp, Cnnm1, Cnot8, Cntn5, Cog4, Col23a1, Col4a2, Col6a2, Cox10, Cpa5, Cpm, Cps1, Crispld2, Cryba1, Cspg4, Ctnna1, Ctnnd2, Dab2ip, Dbf4, Ddx11, Ddx31, Ddx46, Ddx6, Dhrs2, Diap2, Dip2b, Dlg2, Dmd, Dnahc2, Dnahc6, Dnajc6, Dnmbp, Dpy19l3, Dpy19l4, Dpyd, E030003E18Rik, Edil3, Efcab11, Efha1, Efna5, Elmo1, Epb4.1, Epb4.9, Erc2, F11r, Fam20b, Fam227a, Fam228b, Fam73b, Fbln1, Fbxo7, Fhad1, Fhod3, Fmn2, Fmnl2, Fndc3b, Frmd4b, Fstl4, Gabra3, Gabrb1, Gas7, Gatad2a, Glis3, Glra3, Gm11567, Gm11595, Gm11710, Gm11711, Gm12185, Gm13139, Gm18756, Gm5087, Gm8234, Gm960, Gng7, Gnpat, Gon4l, Gpc4, Gpc5, Gpr45, Gprc5b, Gpsm1, Gria1, Grina, Grlf1, Grm5, Gtf2h5, Gys2, Hat1, Hdac4, Heatr7a, Hmbox1, Hmcn1, Hnf4g, Hpse2, Htr1f, Icosl, Ifi47, Ifrd2, Ikzf3, Il1rapl1, Inpp5f, Ip6k1, Iqck, Itga8, Itga9, Jph3, Jsrp1, Kcnq5, Kcnu1, Kif16b, Kif21b, Klra15, Klra22, Kpnb1, Krt78, Ktn1, Lass3, Lhfpl3, Lilra6, Limk1, Lmbr1, Lmx1b, Lrba, Lrguk, Lrrc69, Lyrm7, Lyst, Macrod2, Magi2, Mapk9, Mark1, Me3, Mecom, Med12l, Med13, Med15, Megf11, Mettl8, Mir1195, Mir297-2, Mir3473, Mir466n, Mir669e, Mllt10, Mmd2, Mme, Mprip, Myh15, Myo18a, Myo1e, N28178, Nars2, Nav2, Nbas, Ndufa6, Neb, Nedd4l, Nfatc1, Nlgn1, Nmt2, Nos1ap, Notum, Npepps, Nploc4, Nt5c3l, Ntrk2, Olfr56, Ophn1, Osbpl6, Otx2os1, Pacs1, Pak3, Pax5, Pax7, Pcbp3, Pcdh9, Pcdha1, Pcdha10, Pcdha11, Pcdha12, Pcdha2, Pcdha3, Pcdha4, Pcdha4-g, Pcdha5, Pcdha6, Pcdha7, Pcdha8, Pcdha9, Pcdhac1, Pcnxl2, Pde4d, Pde4dip, Pfas, Pfkfb4, Pgm5, Phactr1, Phex, Phf21a, Pik3r3, Pira1, Pitpnc1, Pkn1, Plcxd2, Plec, Plk1s1, Pls1, Pou2f3, Ppm1l, Ppp2r2b, Ppp3cc, Ppp4r1l-ps, Prrc2b, Prss45, Prss46, Psd3, Psma5, Pter, Ptpn22, Ptprj, Pus10, R3hdm2, Rab30, Rac2, Rad51b, Ralgapb, Rap1gap, Rassf3, Rbfox3, Rbpms, Reln, Rfx4, Rgl1, Rgs17, Rgs6, Ripk3, Rnf150, Rnf41, Rock1, Rock2, Rora, Rptor, Rras2, Rtn4rl1, Runx2, Sacs, Samd4, Sarnp, Scaper, Sdk1, Sec23ip, Sec24d, Sema5a, Serac1, Sh2d3c, She, Sik3, Sim2, Skap1, Slc13a3, Slc18b1, Slc24a2, Slc24a3, Slc25a38, Slc25a40, Slc39a11, Slc39a9, Smg6, Smoc2, Snph, Sorbs1, Spag16, Spag5, Spats2, Spock2, Spon1, Sptbn2, Ssh2, Stat5b, Stpg2, Stx8, Supt3, Suv420h1, Swap70, Syn3, Tada2a, Tanc2, Tbc1d16, Tbc1d22a, Tbl3, Tenm4, Tex14, Thsd4, Thsd7b, Tmc1, Tmcc2, Tmem131, Tmem132c, Tmem135, Tnrc6a, Tnrc6b, Tnrc6c, Traf3, Trex1, Trpm3, Tspan15, Ttc27, Ttc28, Ttn, Txnl1, Ube2f, Ulbp1, Unc13b, Usp53, Utrn, Vav1, Vwf, Wbp1l, Wdfy4, Wfdc2, Wipf2, Wtip, Xirp2, Zbtb20, Zbtb48, Zcchc16, Zfp322a, Zfp407, Zfp423, Zfp641, Zfp862, Zfr2, Znrf3.
