# Supplementary material for: Genome-wide identification of genic and intergenic neuronal DNA regions bound by Tau protein under physiological and stress conditions
Source: Nucleic Acids Res. 2018 Oct 13;46(21):11405–22. doi: 10.1093/nar/gky929 (PMC6265482; doi:10.1093/nar/gky929)
Supplement: Supplementary Data [file gky929_supplemental_files.zip › Table S4.docx]

**Table S4.** Sequences (5’ to 3’) of the different primers used in qPCR assays.

|  | Forward | Reverse |
| --- | --- | --- |
| Polr2a | GCCAGGACACTCGGTCATG | TGCGCACCATCAAGAGAGTG |
| Camk1 | CAAGAGCAAGTGGAAGCAAG | AGAGGTGTGGGGTCAGAAAC |
| Jarid2 | GAAGGCGGTAAATGGGCTTCT | TCGTTGCTAGTAGAGGACACTT |
| Trex1 | CAGCATCTGTCAGTGGAAGCC | GCCAGGGGTGTAGTAGC |
| Grm5 | CCCAGCACAAGTCGGAAATAG | TGTCTGGTTGGGGTTCTCCTT |
| Dlg2 | CTGTCACGAGGCAGGAAATAAA | CGACTTCGTAGTCACGCTTTG |
| Eif2a | CACCGCTGTTGACAGTCAGAG | GCAAACAATGTCCCATCCTTACT |
| Xrcc6 | ATGTCAGAGTGGGAGTCCTAC | TCGCTGCTTATGATCTTACTGGT |
| Dlg1 | CGAAGAGTCACGTCGTTTTGA | TCTCCAAAGCGGAAGTTCAGT |
| Hmbs | TCCCTGAAGGATGTGCCTA | AAGGGTTTTCCCGTTTGC |
| Ppib | GGAGATGGCACAGGAGGAAA | CCGTAGTGCTTCAGTTTGAAGTTCT |
| Rplp0 | CACTGGTCTAGGACCCGAGAAG | GGTGCCTCTGGAGATTTTCG |
