# Supplementary figures and images for: Genome-wide identification of genic and intergenic neuronal DNA regions bound by Tau protein under physiological and stress conditions
Source: Nucleic Acids Res. 2018 Oct 13;46(21):11405–22. doi: 10.1093/nar/gky929 (PMC6265482; doi:10.1093/nar/gky929)

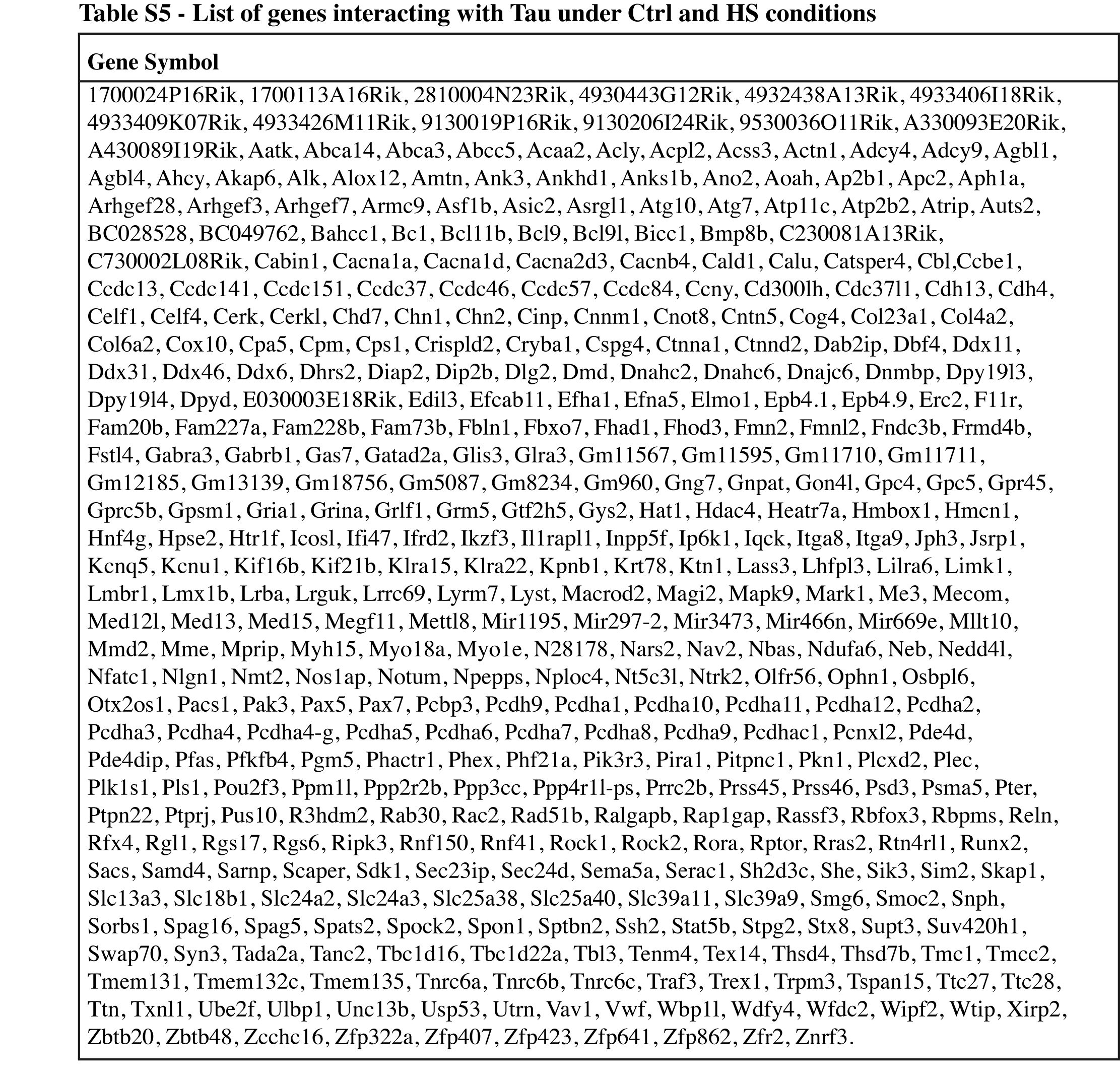

Supplement: Supplementary Data [file gky929_supplemental_files.zip › Table S5.tif]
